# Aging and Strength Training Influence Knee Extensor Intermuscular Coherence During Low- and High-Force Isometric Contractions

**DOI:** 10.3389/fphys.2018.01933

**Published:** 2019-01-23

**Authors:** Simon Walker, Janne Avela, Jan Wikgren, Romain Meeusen, Harri Piitulainen, Stuart N. Baker, Tiina M. Parviainen

**Affiliations:** ^1^Neuromuscular Research Center, Faculty of Sport and Health Sciences, University of Jyväskylä, Jyväskylä, Finland; ^2^Department of Psychology, Centre for Interdisciplinary Brain Research, Faculty of Education and Psychology, University of Jyväskylä, Jyväskylä, Finland; ^3^Human Physiology Research Group, Vrije Universiteit Brussel, Brussels, Belgium; ^4^Department of Neuroscience and Biomedical Engineering, Aalto University, Espoo, Finland; ^5^Institute of Neuroscience, Medical School, Newcastle University, Newcastle upon Tyne, United Kingdom

**Keywords:** alpha-motoneuron, motor control, voluntary contraction, lower-limb, Piper rhythm, beta-band

## Abstract

Aging is associated with reduced maximum force production and force steadiness during low-force tasks, but both can be improved by training. Intermuscular coherence measures coupling between two peripheral surface electromyography (EMG) signals in the frequency domain. It is thought to represent the presence of common input to alpha-motoneurons, but the functional meaning of intermuscular coherence, particularly regarding aging and training, remain unclear. This study investigated knee extensor intermuscular coherence in previously sedentary young (18–30 years) and older (67–73 years) subjects before and after a 14-week strength training intervention. YOUNG and OLDER groups performed maximum unilateral isometric knee extensions [100% maximum voluntary contraction (MVC)], as well as force steadiness tests at 20 and 70% MVC, pre- and post-training. Intermuscular (i.e., EMG-EMG) coherence analyses were performed for all (three) contraction intensities in vastus lateralis and medialis muscles. Pre-training coefficient of force variation (i.e., force steadiness) and MVC (i.e., maximum torque) were similar between groups. Both groups improved MVC through training, but YOUNG improved more than OLDER (42 ± 27 Nm versus 18 ± 16 Nm, *P* = 0.022). Force steadiness did not change during 20% MVC trials in either group, but YOUNG demonstrated increased coefficient of force variation during 70% MVC trials (1.28 ± 0.46 to 1.57 ± 0.70, *P* = 0.01). YOUNG demonstrated greater pre-training coherence during 20% and 70% MVC trials, particularly within the 8–14 Hz (e.g., 20%: 0.105 ± 0.119 versus 0.016 ± 0.009, *P* = 0.001) and 16–30 Hz (20%: 0.063 ± 0.078 versus 0.012 ± 0.007, *P* = 0.002) bands, but not during 100% MVC trials. Strength training led to increases in intermuscular coherence within the 40–60 Hz band during 70% MVC trials in YOUNG only, while OLDER decreased within the 8–14 Hz band during 100% MVC trials. Age-related differences in intermuscular coherence were observed between young and older individuals, even when neuromuscular performance levels were similar. The functional significance of intermuscular coherence remains unclear, since coherence within different frequency bands did not explain any of the variance in the regression models for maximum strength or force steadiness during 20 and 70% MVC trials.

## Introduction

Aging is associated with degenerations in neural functioning that reduce performance during voluntary force production tasks. For example, force steadiness during low- and moderate-force isometric contractions is poorer in older individuals (Tracy and Enoka, 2006; Griffin et al., 2009) and particularly so in those with a history of falls (Carville et al., 2007). This is accompanied by less (larger) motor neurons/units (Lexell et al., 1988), which increases the size of each motor unit (Piasecki et al., 2016), and by greater variability in motor unit discharge rates (Tracy et al., 2005; Kallio et al., 2012). Strength training, which is typically used to combat the deleterious effects of aging on strength and muscle mass, improves activation of motor units during high-force tasks in both young and older individuals (Häkkinen and Häkkinen, 1995; Van Cutsem et al., 1998; Kamen and Knight, 2004; Walker et al., 2014). However, the processes within the neural circuitry that reduce variability in motor unit discharge rates and generate increased force output remain unknown.

One approach in neurophysiology is to compute coherence between two biological signals. Coherence is a measure of correlation between two signals in the frequency domain (Halliday et al., 1995). Coherence analysis estimates the amount of common neural input between two sites during voluntary motor tasks (Ushiyama and Ushiba, 2013). Corticomuscular coherence has been assessed between signals representing cortical (by electroencephalography or magnetoencephalography) and muscular [by electromyography (EMG)] activities. Alternatively, intermuscular (EMG–EMG) coherence between two muscles’ activities has been assessed. Broadly, intermuscular coherence may reflect shared neural inputs from cortical, subcortical and spinal influences (Grosse et al., 2002). Although not explicitly known, coherence within the 8–14 Hz frequency band may be related to Ia afferent feedback (Lippold, 1970), although somatosensory feedback to the cortex appears to operate at <3 Hz (Bourguignon et al., 2017). Coherence within the 15–30 Hz frequency band suggests common corticospinal input (Fisher et al., 2012) potentially driven by sensorimotor cortex oscillations (Bourguignon et al., 2017), while 40–60 Hz frequency band has potentially reticulospinal origin (Garcia-Rill et al., 2016). Intermuscular coherence is relatively easy to measure and compute for large populations (Jaiser et al., 2016) and may have clinical relevance (Fisher et al., 2012; Larsen et al., 2017). Furthermore, intermuscular coherence methods allow examination of high-force contractions (>70% of maximum), whereas EEG signals may be contaminated by muscular artifacts when examining high-force corticomuscular coherence.

Since corticomuscular coherence is most robust within 15–30 Hz band during steady low-force isometric contraction (Conway et al., 1995; Baker et al., 1997; Salenius et al., 1997), scientific investigation has predominantly focused on these frequencies. Coherence at 15–30 Hz has been suggested to be related to a neural strategy for precision tasks, reflecting a contribution from the corticospinal tract (Fisher et al., 2012). Concurrently, it has long been known that voluntary contractions can exhibit oscillations at higher frequencies, e.g., the so-called Piper rhythm at 40–60 Hz (Piper, 1907). Corticomuscular coherence appears to be force-dependent, since higher force contractions demonstrate greater Piper coherence (Brown et al., 1998; Mima et al., 1999). Summarizing several studies’ findings, it appears that contraction intensities above 60% of maximum voluntary contraction (MVC) are required to observe this force-related increase in 40–60 Hz coherence, although this is not present in all muscles (Ushiyama et al., 2012). Since 15–30 Hz corticomuscular coherence has been shown to be lower in trained individuals (weightlifters and ballet dancers) compared to untrained individuals (Ushiyama et al., 2010), it would be of interest to determine whether strength training influences 40–60 Hz coherence in a contrasting fashion.

However, the intermuscular coherence method has come under scrutiny as to its ability to determine the amount of common neural input due to the potentially decorrelating influence of multiple sources of common synaptic input (Farina et al., 2014). Hence, it is of interest to determine whether intermuscular coherence can distinguish between groups and possible intervention effects if this method is to gain more widespread usage. Consequently, the present study aimed to determine; (1) the magnitude of intermuscular coherence during low- and high-force isometric knee extensions in healthy young and older adults and (2) possible short-term strength training-induced changes in intermuscular coherence during low- and high-force contractions in young and older adults. In order to estimate potential functional significance of coherence, regression models were constructed to test whether intermuscular coherence strengths within 2–6, 8–14, 16–30, and 40–60 Hz frequency bands predicted force steadiness and/or maximum force production during the isometric contractions. Regression models were assessed for; (1) both pre-training force steadiness and maximum force values and (2) the changes in coherence and force steadiness/maximum force due to strength training.

## Materials and Methods

### Subjects

Sixteen young (YOUNG, age range: 18–31, 4 men and 12 women) and 16 older (OLDER, age range: 66–73, 8 men and 8 women) individuals volunteered for the study. None of the subjects had any history of neurological conditions including diabetes mellitus, none took any neurotropic medication, and none had any lower limb injuries or disabilities. Additionally, all older subjects underwent a medical examination to ensure there were no contraindications to performing maximal effort testing and strength training.

One young and three older subjects did not complete the strength training intervention for various reasons and dropped-out of the study (illness not related to the study, loss of interest, one older man suffered injury during the training). Significant intermuscular coherence across a broad frequency spectrum was observed in 13 out of 15 YOUNG and 11 out of 13 OLDER that completed 14 weeks of strength training. These subjects were then taken forward into subsequent analyses. The final characteristics of the subjects in both age groups were as follows: YOUNG: age 25 ± 4 years, height 170 ± 10 cm, body mass 64 ± 11 kg, body mass index 22 ± 4 (3 men and 10 women), OLDER: age 69 ± 3 years, height 166 ± 9 cm, body mass 72 ± 12 kg, body mass index 26 ± 3 (5 men and 6 women).

All subjects provided written informed consent prior to the study. The study was approved by the ethical committee of the University of Jyväskylä and was performed according to the Declaration of Helsinki.

### Test Procedures

Prior to testing, the subjects attended a familiarization visit to the lab. The purpose of this session was to individualize the settings of the custom-built isometric dynamometer, teach the correct contraction techniques for maximal and submaximal unilateral isometric knee extension tasks, allow practice trials, and place indelible ink tattoos marking the EMG locations of m. vastus lateralis (VL) and vastus medialis (VM). Subjects performed unilateral knee extension trials with the right leg and a knee angle of 110°. Non-elastic straps about the ankle, knee and hip secured the subjects to the dynamometer and prevented movement during contraction. The subjects’ arms were folded across their chest. They were instructed to be as relaxed as possible in the upper body, and to concentrate on producing force through only extension about the knee.

One week later, subjects reported to the lab for their pre-training tests. Following preparation and placement of EMG electrodes, subjects performed four maximum voluntary contractions (MVC). Subjects were instructed to perform each trial as “hard and as fast as possible” and were given constant verbal encouragement. Real-time visual feedback of force production was provided during all tasks. The best trial (i.e., highest force) was taken as that individual’s MVC performance. Thereafter, force levels equivalent to 20 and 70% of MVC were calculated and displayed as horizontal bars on the screen. Subjects then performed 4 × 30 s contractions at approximately 20% MVC with 60 s rest between trials. Next, subjects performed four sets of 4 × 6 s contractions at approximately 70% MVC with 15 s rest between trials and 90 s rest between sets. During the submaximal trials, subjects were instructed to maintain a constant force level as close as possible to the target (horizontal) bar. Force steadiness was taken as the averaged Coefficient of Variation in force (CV force %) of all trials (CV = SD/mean^∗^100). Finally, the subjects performed 1–2 MVC trials at the end of the testing session to ensure that no fatigue had occurred due to the test procedures (mean change in force from the beginning to the end of the testing was 575 ± 108N to 565 ± 88N, *P* = 0.332).

**FIGURE 1:**
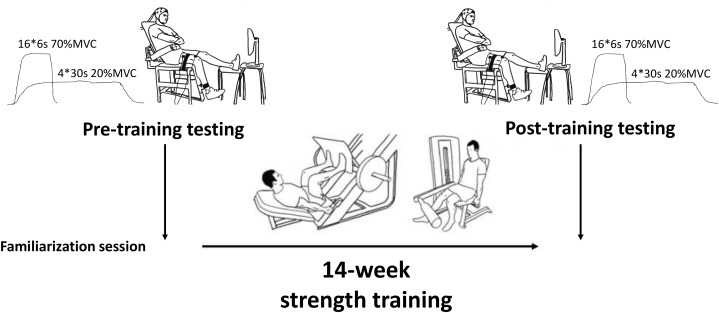
Isometric knee extension experimental setup.

Following these measurements, subjects completed 14 weeks of supervised and progressive strength training in the University gym (details are given below) prior to returning for post-training tests (Figure 1). These tests took place 7 days after the last training session to eliminate any residual fatigue from training, and the test time-of-day was matched to that of the pre-training test (±1 h).

**FIGURE 2:**
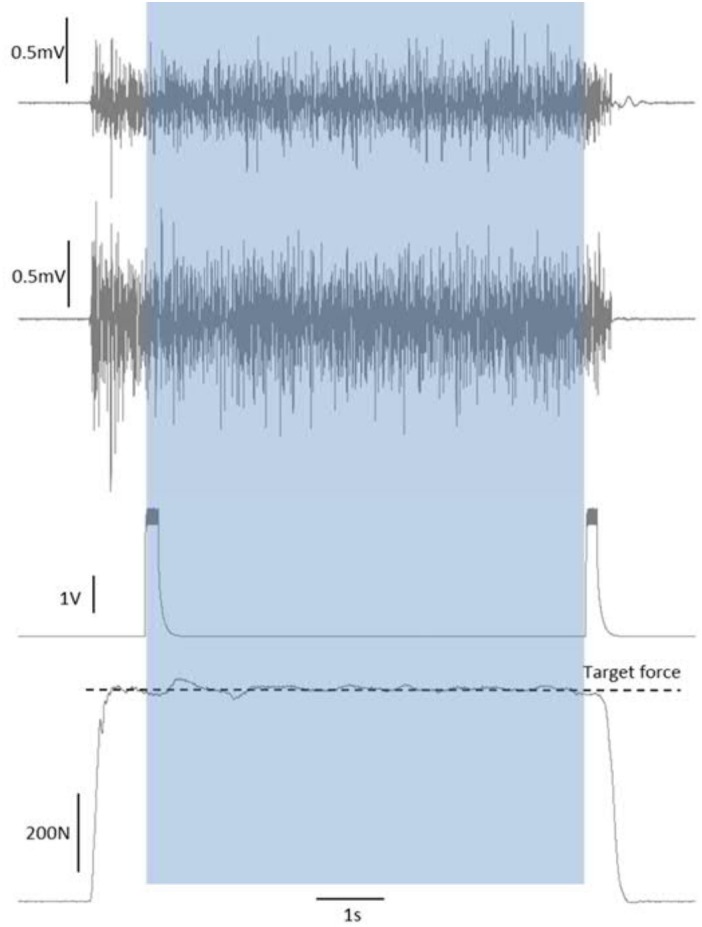
Synchronization of force and EMG signals by start/end of trial square-pulse triggers. The shaded area shows the single-trial duration that was taken forward into the analyses.

During each maximal and submaximal knee extension trial a square-pulse trigger was manually generated to the recording software once the subjects had reached a steady force level. This pulse was used to synchronize the start of the analysis (see later). Upon completion of the contraction time (30 s for 20%, 6 s for 70%, and 3 s for 100% MVC trials), a second square-pulse trigger was manually generated to synchronize the end of the trial/analyses, and then the subject was instructed to relax (Figure 2).

### Electromyography Recordings

Following shaving and skin preparation, bipolar Ag/AgCl electrodes (5 mm diameter, 20 mm inter-electrode distance) were positioned on VL and VM of the right leg according to SENIAM guidelines. Placement was in-line with the orientation of the underlying fascicles, and guided by the tattoos to ensure reproducibility from session to session. Raw EMG signals were amplified at a gain of 500 (bandwidth 10–500 Hz, common mode rejection ratio >100 dB, input impedance >100 MΩ, baseline noise <1 μV rms) and sampled at 2000 Hz. Raw signals were sent from a hip-mounted pack to a receiving box (Telemyo 2400R, Noraxon, Scottsdale, United States), then were relayed to an analog-to-digital converter (Micro1401, Cambridge Electronic Design, United Kingdom) and recorded by Signal 4.10 software (Cambridge Electronic Design, United Kingdom).

### Intermuscular Coherence Analyses

Coherence analyses were performed using Matlab (Mathworks, Natick, MA, United States) using customized scripts in a similar manner as previously reported (Jaiser et al., 2016). Raw data was visually inspected, and in the rare case that single trials were performed incorrectly or demonstrated signal disruption or excessive noise, they were removed from the analyses. Analysis used the time window between the start and end triggers described above (∼30 s for each of the four 20% MVC trials, ∼6 s for each of the sixteen 70% MVC trials, and ∼3 s for the four MVC trials). Trials of the same task were collated to yield total recording lengths of ∼120, 96, and 12 s for the 20, 70, and 100% MVC tasks, respectively. EMG signals were full-wave rectified prior to analyses. Available data was separated into non-overlapping windows 1024 samples in length, which were subjected to fast Fourier transform. This gave a frequency resolution of 1.95 Hz.

Denoting the Fourier transform of the two signals in the l’th window at frequency λ as *F*_1,*l*_(λ) and *F*_2,*l*_(λ), the auto-spectrum of the first signal was given by:

$$f_{11}(\lambda )=\frac{1}{L}\overset{L}{\underset{l=1}{\Sigma }}F_{1},l\left(\lambda \right)F_{1},l\left(\lambda \right)*$$

The cross-spectrum was calculated as:

$$f_{12}(\lambda )=\frac{1}{L}\overset{L}{\underset{l=1}{\Sigma }}F_{1},l\left(\lambda \right)F_{2},l\left(\lambda \right)*$$

Here ^∗^ denotes the complex conjugate, and *L* is the total number of sections. The pre-training *L*-values were as follows; intermuscular coherence 20% = 226 ± 2, 70% = 179 ± 4, 100% = 28 ± 2 (post-training values were similar).

Coherence was calculated as the cross-spectrum normalized by the auto-spectrum:

$$C(\lambda )=\frac{{\left|f_{12}\left(\lambda \right)\right|}^{2}}{f_{11}(\lambda )f_{22}\left(\lambda \right)}$$

coherence above *Z* was considered significantly above chance, according to the formula developed by Brillinger (1981) and given by Rosenberg et al. (1989):

$$Z=1-\alpha^{1/\left(L-1\right)}$$

where the significance level α was set to 0.05.

Intermuscular coherence spectra for 0–100 Hz were averaged across subjects in each age group (i.e., YOUNG and OLDER). The group significance level was then determined using the method of Evans and Baker (2003). Thereafter, coherence was sectioned into 2–6, 8–14, 16–30, and 40–60 Hz bands and the average coherence within each window used to compare between groups and time points.

### Body Composition Measurements

On a separate occasion, participants visited the lab after a 12-h overnight fast. After determination of height by a fixed wall-mounted scale, participants underwent full body scanning by dual-energy X-ray absorptiometry (DXA) in minimal clothing (LUNAR Prodigy Advance with encore software version 9.3, GE Medical Systems, United States). The legs were separated by a polystyrene block and secured by inelastic straps about the ankles to ensure no movement during the scanning and accurate replacement after the training period. An operator-defined Range-of-Interest for the thigh region was manually produced from anatomical landmarks; the same operator performed all analyses. The proximal and distal ends of the Range-of-Interest were the apex of the greater trochanter and knee joint space, respectively. Thigh region fat mass and fat-free mass was determined from the scans to assess morphological adaptations to strength training.

### Strength Training

Subjects reported to the University gym twice per week on non-consecutive days – typically Monday/Thursday or Tuesday/Friday. Each training session consisted of around 9 exercises for all muscle groups (i.e., whole body training), however, all leg exercises were performed first since this was the primary target muscle group. The specific training program is given in Table 1. Loads progressed incrementally throughout the training period and the subjects were encouraged to lift the load until momentary failure [i.e., repetition maximum (RM)]. In the present study, subjects performed sets of 16 RM in the beginning of training progressing to sets of 6 RM at the end of the training period. Lighter load sets were included toward the end of the training period, in which the concentric phase was performed with maximum velocity but momentary failure was not realized. In general, this type of strength training program can be considered to be linearly periodized, progressing from muscular endurance-focused to hypertrophy- and maximum strength-focused training, and finishing with power-focused training. Subjects were instructed how to perform each exercise and technique was constantly monitored by qualified instructors. The tempo for muscular endurance and hypertrophy trainings was 2 s for concentric and 2 s for eccentric phases, while tempo for maximum strength and power trainings was as fast as possible for concentric and either as fast as possible or 2 s for eccentric phases depending on the type of exercise (noted in Table 1). Subjects were allowed to continue their habitual physical activities, such as low intensity walking, cycling, and swimming at a frequency of 1–3 times per week, during the study period.

**Table 1:** Progressive strength training program completed by all subjects.

Weeks	Main training goal	Session	Exercise	Sets	Reps	%1-RM	Tempo	Inter-set rest
1–2	Muscular endurance	1	Leg press	3	14–16	40–60%	2:2	60 s
			Knee extension	2	14–16	40–60%		
			Knee flexion	2	14-16	40–60%		
			Chest press	2	14–16	40–60%		
			Lat pulldown	2	14–16	40–60%		
			Triceps extension	2	14–16	40–60%		
			Ab curl	2	16–20	BM		
			Back extension	2	16–20	BM		
								
		2	Leg press	3	14–16	40–60%	2:2	60 s
			Knee extension	2	14–16	40–60%		
			Knee flexion	2	14–16	40–60%		
			Shoulder press	2	14–16	40–60%		
			Seated row	2	14–16	40–60%		
			Biceps curl	2	14–16	40–60%		
			Seated calf-raise	2	14–16	40–60%		
			Ab curl	2	16–20	40–60%		
			Back extension	2	16–20	40–60%		
								
3–5	Hypertrophy	1	Leg press	3	10–12	70–80%	2:2	60 s
			Knee extension	3	10–12	70–80%		
			Knee flexion	2	10–12	70–80%		
			Chest press	3	10–12	70–80%		
			Lat pulldown	2	10–12	70–80%		
			Triceps extension	2	10–12	70–80%		
			Standing calf-raise	3	10–12	70–80%		
			Ab curl	2	14–16	50–70%		
			Back extension	2	14–16	50–70%		
								
		2	Leg press	3	10–12	70–80%	2:2	60 s
			Knee extension	2	10–12	70–80%		
			Knee flexion	3	10–12	70–80%		
			Shoulder press	2	10–12	70–80%		
			Seated row	3	10–12	70–80%		
			Biceps curl	2	10–12	70–80%		
			Seated calf-raise	3	10–12	70–80%		
			Ab curl	2	14–16	50–70%		
			Back extension	2	14–16	50–70%		
								
6–7	Hypertrophy	1	Leg press	4	8–10	80–85%	2:2	60 s
			Knee extension	3	8–10	80–85%		
			Knee flexion	2	8–10	80–85%		
			Chest press	4	8–10	80–85%		
			Lat pulldown	2	8–10	80–85%		
			Triceps extension	2	8–10	80–85%		
			Standing calf-raise	3	8–10	80–85%		
			Ab curl	2	10–12	70–80%		
			Back extension	2	10–12	70–80%		
		2	Leg press	4	8–10	80–85%	2:2	60 s
			Knee extension	2	8–10	80–85%		
			Knee flexion	3	8–10	80–85%		
			Shoulder press	2	8–10	80–85%		
			Seated row	4	8–10	80–85%		
			Biceps curl	2	8–10	80–85%		
			Seated calf-raise	3	8–10	80–85%		
			Twisting Ab	2	10–12	70–80%		
			Back extension	2	10–12	70–80%		
8–9	Maximum strength	1	Leg press	3	5–8	85–90%	2:2	120 s
			Knee extension	3	6–8	85–90%		
			Knee flexion	3	6–8	85–90%		
			Lunge^∗^	2	12–14	60–70%		
			Standing calf-raise	2	12–14	60–70%		
			Seated calf-raise	2	8–10	80–85%		
			Bench press^∗^	2	12–14	60–70%		
			Db shoulder press^∗^	2	12–14	60–70%		
			Ab crunch	2	12–14	BM		
			Back extension	2	12–14	BM		
								
		2	Smith-machine squat	3	5–8	85–90%	2:2	120 s
			Knee extension	3	6–8	85–90%		
			Knee flexion	3	6–8	85–90%		
			Db deadlift^∗^	2	12–14	60–70%		
			Standing calf-raise	4	12–14	60–70%		
			Bent-over row^∗^	2	12–14	80–85%		
			Assisted pull-up	2	12–14	60–70%		
			Ab crunch	2	12–14	BM		
			Back extension	2	12–14	BM		
								
10–12	Maximum strength and power	1	Leg press	3	4–6	90–95%	1:2	120 s
			Knee extension	3	6–8	85–90%	1:2	
			Knee flexion	3	6–8	85–90%	1:2	
			Standing calf-raise	3	7–8	85–90%	1:2	
			Calf jumps^∗^	1	7–8	BM	0:0	
			Lunge jumps^∗^	2	5	BM	0:2	
			Bench press^∗^	3	10–12	70–80%	2:2	
			Db shoulder press^∗^	2	10–12	70–80%	2:2	
			Ab crunch	2	14–16	BM	2:2	
			Back extension	2	14–16	BM	2:2	
								
		2	Smith-machine squat	3	4–6	90–95%	1:2	120 s
			Knee extension	3	6–8	85–90%	1:2	
			Knee flexion	3	6–8	85–90%	1:2	
			Standing calf-raise	3	7–8	85-90%	1:2	
			Calf jumps^∗^	1	7–8	BM	0:0	
			CMJ^∗^	2	5	BM	0:2	
			Bent-over row^∗^	3	10–12	70–80%	2:2	
			Assisted pull-up	2	10–12	70–80%	2:2	
			Ab crunch	2	14–16	BM	2:2	
			Back extension	2	14–16	BM	2:2	
13–14	Power	1	Leg press	3	4–6	50–60%	0:2	120 s
			Knee extension	3	4–6	90–95%	1:2	
			Knee flexion	3	4–6	90–95%	1:2	
			Standing calf-raise	1	7–8	85–90%	1:2	
			Calf jumps^∗^	3	7–8	BM	0:0	
			Lunge jumps^∗^	3	5	BM	0:2	
			Pec Deck	3	8–10	80–85%	2:2	
			Assisted dips^∗^	3	8–10	80–85%	2:2	
			Ab crunch	2	16–20	BM	2:2	
			Back extension	2	16–20	BM	2:2	
		2	Smith-machine squat	3	4–6	50–60%	0:2	120 s
			Knee extension	3	4–6	90–95%	1:2	
			Knee flexion	3	4–6	90–95%	1:2	
			Standing calf-raise	1	7–8	85–90%	1:2	
			Calf jumps^∗^	3	7–8	BM	0:0	
			CMJ^∗^	3	5	BM	0:2	
			Seated row	3	8–10	80–85%	2:2	
			Upright row^∗^	3	8–10	80–85%	2:2	
			Ab crunch	2	16–20	BM	2:2	
			Back extension	2	16–20	BM	2:2	


**Tempo refers to the time of the concentric:eccentric phase of the lift in seconds. 1-RM, one-repetition maximum; Ab, abdominal; Db, dumbbell; BM, body mass; CMJ, countermovement jump; ^∗^, free-weight exercise*.*

### Statistical Methods

Results are reported as means and standard deviations (SD). To compare coherence-frequency curves both within- and between-groups, *Z*-scores via a hyperbolic arctan transform were generated according to the methods of Jaiser et al. (2016). Individual subject’s data were inspected and only subjects showing significant broad-spectra coherence were included in this study. Statistically significant differences for each frequency bin, based on the *Z*-score analysis, was estimated using Monte-Carlo simulations and are highlighted in Figures 4 and 6.

Thereafter, coherence within each frequency band was also assessed statistically within- and between-groups using SPSS software version 24 (IBM, New York, NY, United States). Similar results were obtained using peak and averaged coherence in each of the frequency bands. For clarity, only average coherence results are reported here. Average coherence values across the various frequency bands were not normally distributed and so the coherence data were log 10 transformed to allow parametric tests to be performed. Repeated measures ANOVA (2 time × 2 group) was used on maximum strength and force steadiness, as well as average coherence across the four frequency bands. *Post hoc* tests were performed with Bonferroni adjustments.

Linear multiple regression analysis was performed using the following predictors; average intermuscular coherence in 2–6, 8–14, 16–30, and 40–60 Hz frequency bands, fat-free mass and fat mass to predict force steadiness and/or maximum force production using the stepwise method in SPSS.

## Results

### Maximum Strength and Force Steadiness Pre- and Post-training

Pre-training 100% MVC torque (YOUNG: 195 ± 53 Nm, OLDER: 178 ± 53 Nm) did not differ between groups. Similarly, pre-training force steadiness did not differ between the age groups during either 20% MVC trials (YOUNG: 0.35 ± 0.15%, OLDER: 0.28 ± 0.09%) or 70% MVC trials (YOUNG: 1.28 ± 0.46%, OLDER: 0.96 ± 0.34%).

Significant main effects for time (*F* = 38.2, *P* < 0.001) and time × group (*F* = 6.1, *P* = 0.022) were observed in MVC. Strength training led to an increase in MVC in both groups (YOUNG: 195 ± 53 Nm to 236 ± 54 Nm, *P* = 0.001; OLDER: 178 ± 53 Nm to 196 ± 49 Nm, *P* = 0.005). However, the improvements obtained by YOUNG were significantly greater than those obtained by OLDER (42 ± 27 Nm versus 18 ± 16 Nm, *P* = 0.022).

**FIGURE 3:**
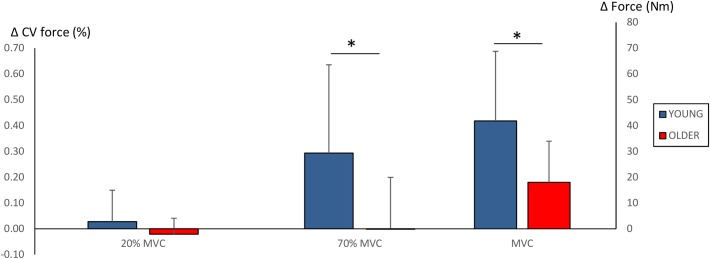
Changes (Δ, mean ± SD) in force steadiness (CV of force %) during 20 and 70% MVC trials and maximum force production (Nm) during 100% MVC trials. ^∗^*P* < 0.0125 between-groups comparisons. Note that increased CV of force % (i.e., greater force fluctuation) reflects poorer force steadiness.

No main effects for force steadiness during 20% MVC trials were observed (YOUNG: 0.35 ± 0.15% and 0.37 ± 0.20%; OLDER: 0.28 ± 0.09% and 0.26 ± 0.07%). However, significant main effects for time (*F* = 5.6, *P* = 0.027), time × group (*F* = 5.8, *P* = 0.025) and group (*F* = 5.8, *P* = 0.025) were observed in force steadiness during 70% MVC trials. YOUNG demonstrated significant worsening of force steadiness during 70% MVC trials (1.28 ± 0.46% to 1.57 ± 0.70%, *P* = 0.01), and the change was statistically different compared to that in OLDER (0.29 ± 0.34% versus -0.02 ± 0.2%, *P* = 0.025). Changes in maximum force production and force steadiness are shown in Figure 3.

### Effect of Aging on Intermuscular Coherence

**FIGURE 4:**
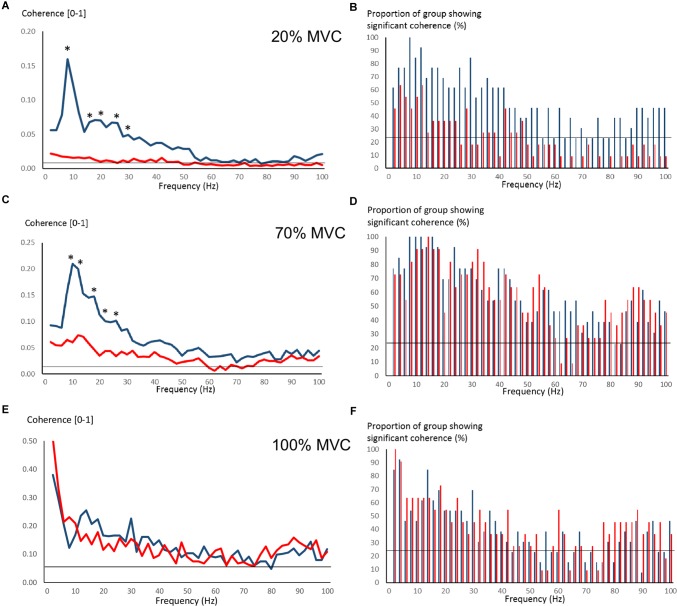
Pre-training intermuscular coherence over 0–100 Hz during 20% **(A)**, 70% **(C)**, and 100% **(E)** MVC trials. Data are averaged for each age-group (YOUNG = blue color, OLDER = red color). ^∗^*P* < 0.05 YOUNG vs. OLDER based on *Z*-score analyses. The accompanying histograms for the three contraction intensities **(B,D,F)** show the proportion of subjects within each age-group demonstrating significant coherence for each frequency bin over 0–100 Hz. The horizontal lines in the figures represent the level of significance.

Figure 4 shows pre-training intermuscular coherence over 0–100 Hz during 20, 70, and 100% MVC trials. Both groups showed relatively large coherence levels over approx. 0–60 Hz, with the exception of OLDER during 20% MVC trials. Significant coherence was a robust finding across a majority of subjects (Figures 4B,D,F). Using *Z*-score comparisons, YOUNG demonstrated significantly larger intermuscular coherence compared to OLDER over frequencies of approximately 8–36 Hz during both 20 and 70% MVC trials (*P* < 0.05, Figures 4A,C). There were no differences between age-groups during 100% MVC trials (Figure 4E).

**FIGURE 5:**
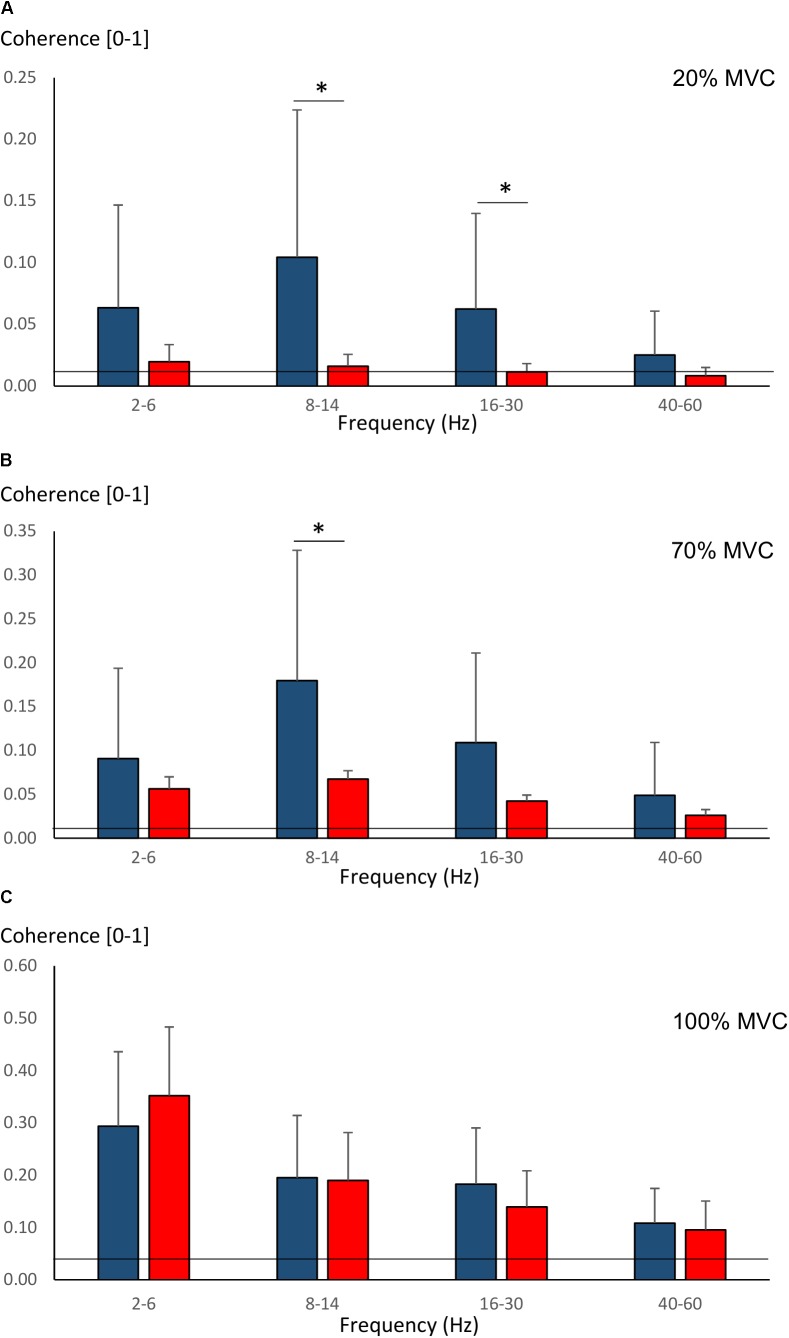
Pre-training average intermuscular coherence (mean ± SD) within each studied frequency band in YOUNG (blue color) and OLDER (red color) during 20% **(A)**, 70% **(B)**, and 100% **(C)** MVC trials. ^∗^*P* < 0.0125.

Pre-training average coherence within the frequency bands 2–6, 8–14, 16–30, and 40–60 Hz is shown in Figure 5. Significant main effects for group were observed in 8–14, 16–30, and 40–60 Hz coherence during 20% MVC trials (*F* = 5.2–14.6, *P* = 0.032–0.001) and in 8–14 and 16–30 Hz coherence during 70% MVC trials (*F* = 11.5, *P* = 0.003). YOUNG demonstrated significantly greater coherence levels than OLDER in the 8–14 and 16–30 Hz bands during 20 and 70% MVC trials pre- (Figures 5A,B) and post-training (not shown).

### Effect of Strength Training on Intermuscular Coherence

**FIGURE 6:**
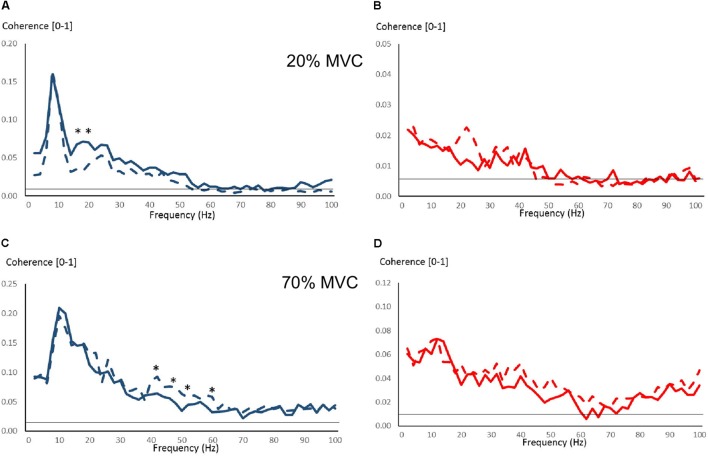
Intermuscular coherence pre- and post-training over 0–100 Hz in YOUNG (blue color) and OLDER (red color) during 20% **(A,B)** and 70% **(C,D)** MVC trials. Data are averaged (Pre-training = solid lines, Post-training = dashed lines). The horizontal lines represent the level of significance for coherence. ^∗^*P* < 0.05 pre- vs. post-training based on *Z*-score analyses.

Few changes were observed due to strength training in intermuscular coherence over 0–100 Hz during 20 and 70% MVC trials (Figure 6). During 20% MVC trials, YOUNG had significantly reduced coherence at 18–20 Hz post-training (*P* < 0.05, Figure 6A). Whereas during 70% MVC trials, YOUNG showed increased intermuscular coherence at 22–24 Hz and over several frequencies of approximately 36–64 Hz (*P* < 0.05, Figure 6C). Sporadic significant, but non-systematic, increases were observed in some frequency bins during 100% MVC in YOUNG (not shown). OLDER showed no training-induced changes in coherence level during any contraction intensity.

**FIGURE 7:**
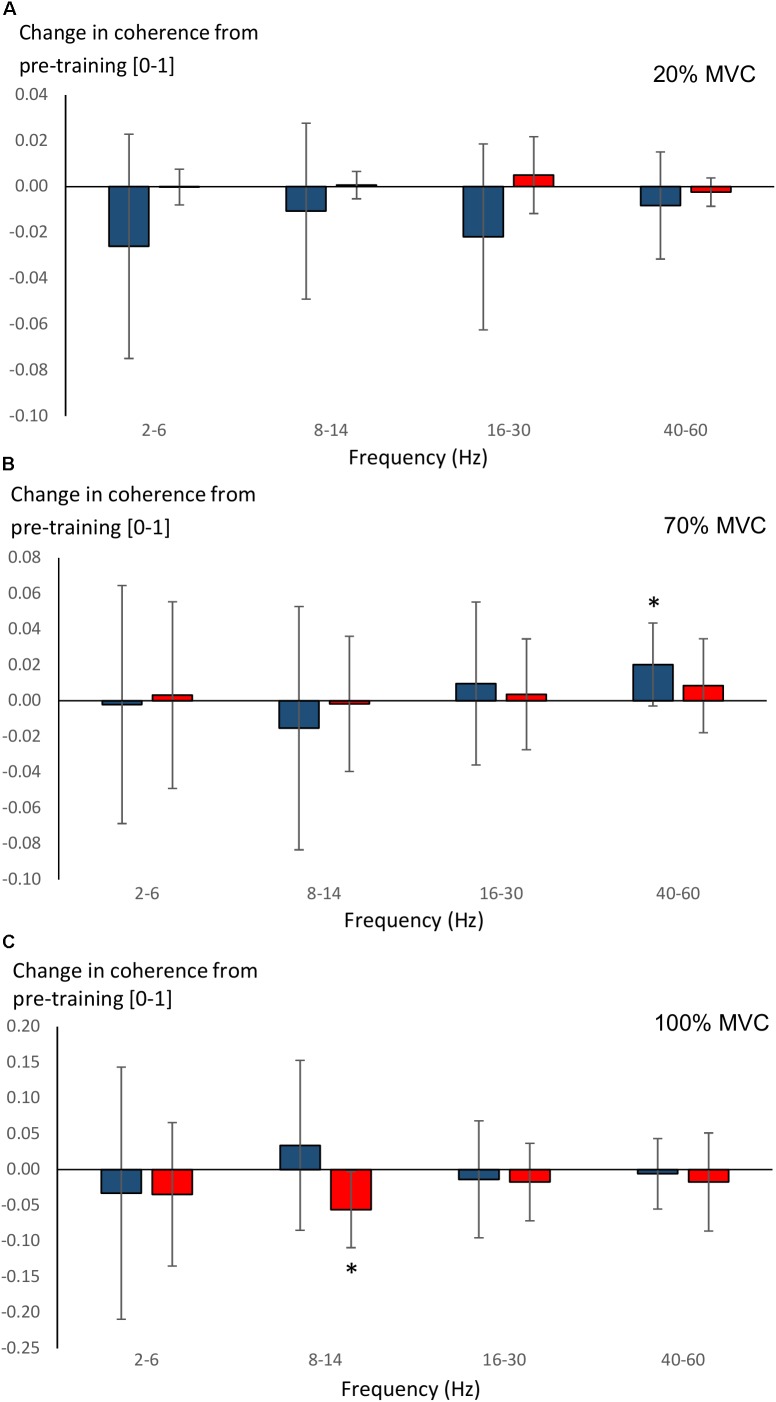
Changes (Δ, mean ± SD) from pre-training in average intermuscular coherence within each studied frequency band in YOUNG (blue color) and OLDER (red color) during 20% **(A)**, 70% **(B)**, and 100% **(C)** MVC trials pre-training. ^∗^*P* < 0.0125 within-group comparisons.

Significant main effects for time (*F* = 8.5, *P* = 0.008) and time × group (*F* = 4.6, *P* = 0.043) were observed in 40–60 Hz coherence during 70% MVC trials. YOUNG significantly increased coherence during 70% MVC trials within the 40–60 Hz band (*P* = 0.008, Figure 7B). During 100% MVC trials, a significant main effect for time × group (*F* = 9.7, *P* = 0.005) was observed in 8–14 Hz coherence. Here, OLDER significantly decreased 8–14 Hz band intermuscular coherence (*P* = 0.003, Figure 7C).

### Body Composition Pre- and Post-training

There were no between-group differences in thigh fat mass, thigh fat-free mass or the change in these variables between-groups. A significant main effect for time (*F* = 44.2, *P* < 0.001) was observed in thigh fat-free mass. Thigh fat-free mass increased in YOUNG (6.4 ± 1.1 to 6.8 ± 1.3 kg, 6 ± 5%, *P* = 0.001) and in OLDER (5.5 ± 1.1 to 5.9 ± 1.1 kg, 7 ± 4%, *P* = 0.001) pre- to post-training.

### Predicting Maximum Strength and Force Steadiness

Pre-training, a significant regression model was observed for 100% MVC torque (*F* = 32.32, *P* < 0.001) explaining 58% of the variance (adjusted *R*^2^ = 0.577). The only significant variable within the model was thigh fat-free mass (beta 0.771, *P* < 0.001). For force steadiness, a significant regression model was observed during 70% MVC trials explaining 40% of the variance (*F* = 16.39, *P* = 0.001, adjusted *R*^2^ = 0.401). The only predictor was 100% MVC torque (beta 0.653, *P* = 0.001). Similar regression models were observed for post-training variables, but no significant models were observed for changes from pre- to post-training.

## Discussion

The present study demonstrated distinctive age-related differences in intermuscular coherence between young and older individuals. Broadly, young individuals demonstrated greater 8–14 and 16–30 Hz intermuscular coherence during 20 and 70% MVC trials. These results suggest differences in the function of the neuronal sensorimotor circuits between the age groups, despite their similar pre-training motor performances (i.e., maximum force production and force steadiness). During 70% MVC trials, young individuals displayed increased 40–60 Hz intermuscular coherence after the training period. However, regression analyses revealed that neither intermuscular coherence magnitude nor training-induced changes in intermuscular coherence could explain the variance in maximum force or force steadiness. Furthermore, YOUNG improved maximum force production to a greater extent than OLDER but showed no training-induced changes in coherence level during 100% MVC. Thus, our results cannot confirm functional significance of intermuscular coherence during maximum force production or force steadiness contractions of the knee extensors within the confinements of our study protocol.

### Effects of Aging

Pre-training maximum isometric force (MVC) did not differ between YOUNG and OLDER in the present study. This may have been due to the subject composition of the two groups, with men accounting for 46% of OLDER and only 30% of YOUNG. Given that thigh fat-free mass was not different between groups, it would be doubtful that specific tension would differ greatly between the groups in our sample. Despite a lack of difference in maximum force production and subsequent force steadiness, YOUNG demonstrated greater intermuscular coherence during 20 and 70% MVC trials across 8–36 Hz frequencies and within 8–14 and 16–30 Hz bands when averaged. This supports the interpretation that difference in coherence reflected central, rather than purely peripheral processes.

Regarding intermuscular coherence within the 16–30 Hz band, it has been shown that the magnitude of coherence is dependent upon an intact (Fisher et al., 2012) and fully functional (Velazquez-Perez et al., 2017) corticospinal tract, and is reduced following acute stroke (Larsen et al., 2017). Consequently, coherence within the 16–30 Hz band can be considered to represent common cortical inputs to motoneurons via the corticospinal tract. The greater 16–30 Hz coherence in intermuscular of YOUNG supports this viewpoint, and suggests that OLDER had compromised corticospinal tract function or a reliance on other neural sites of modulation to regulate performance. For example, one possible reason for compromised corticospinal tract function would be the well-documented loss of myelinated corticospinal axons (Terao et al., 1994) as well as neurons throughout the central nervous system (Marner et al., 2003).

Our results are in direct contrast to the corticomuscular coherence results obtained by Kamp et al. (2013), who suggested that greater coherence was necessary during healthy aging as a means of counteracting loss of cortical functioning. However, subjects in the study by Kamp et al. (2013) performed pseudo-isometric wrist extension contractions. In needing to maintain a constant wrist position in a free environment, the subjects may have had to rely on proprioception to a greater extent than during a typical isometric contraction. We have recently shown that corticokinematic coherence, i.e., coherence between sensorimotor cortex (MEG) and foot acceleration signals during repetitive passive ankle rotations, is greater in older subjects likely due to inefficient cortical processing of proprioceptive afference (Piitulainen et al., 2018). It is important to note that different coherence measures along with varying experimental conditions reflect different aspects of sensorimotor processing/functioning. Thus, the findings should be discussed within the methodological constraints of that particular study.

The present study’s lack of difference in force steadiness between age-groups is not entirely surprising considering that the muscle group tested was the knee extensors and that the lowest force level was 20% of MVC (Enoka et al., 2003). Typically, differences in force steadiness between young and older individuals would be observable more clearly in finger muscles and at force levels ≤10% of MVC (Enoka et al., 2003). Nevertheless, YOUNG demonstrated greater intermuscular coherence during our lowest contraction intensity (i.e., 20% MVC trials) within 8–14 and 16–30 Hz bands. Intermuscular coherence is thought to reflect common neural input to motoneurons from cortical, subcortical and spinal influences (Grosse et al., 2002). Therefore, the present study’s findings suggest that aging reduces synchrony of common neural inputs to alpha-motoneurons.

While the source of 16–30 Hz coherence has been discussed above, intermuscular coherence around 10 Hz has been proposed to represent the level of Ia afferent feedback (Lippold, 1970; Erimaki and Christiakos, 2008). Previous studies have observed differences in the ability of young and older individuals to modulate force control via afferent feedback (Baudry et al., 2010; Holmes et al., 2015). Hence, it is plausible that our finding of lower 8–14 Hz intermuscular coherence during both 20 and 70% MVC trials in OLDER compared to YOUNG reflects a reduced ability to modulate force via afferent feedback. If older individuals have compromised afferent feedback, which is reflected in the level of 8–14 Hz intermuscular coherence, the question remains as to what mechanisms older individuals use to compensate for this in order to perform at a similar level to young. One other possibility is that older individuals had several common inputs within the 8–14 and 16–30 Hz frequency bands; the superimposition of these multiple sources could lead to lower coherence levels (Farina et al., 2014).

There are several limitations that should be acknowledged when utilizing intermuscular coherence measures. For example, when comparing between individuals, the EMG signals and thus its spectral content vary due to both anatomical and morphological reasons. Even individual motor unit action potentials have different shapes. One possibility is that electrodes placed close to the innervation zone may show lower coherence (Keenan et al., 2011). Innervation zone location can be highly variable along the muscle belly between individuals, and the zones are known to shift with force level even during isometric contractions (Piitulainen et al., 2009). Nevertheless, EMG locations were identified by SENIAM guidelines and they were as identical as possible for all individuals, hence, a systematic difference in placement relative to innervation zone seems unlikely, particularly since the focus of SENIAM is to place electrode between the musculotendinous junction and innervation zone. Further, one study showed no influence of electrode location relative to the innervation zone on corticomuscular coherence (Piitulainen et al., 2015). Hence, these potential influences are perhaps minor.

Another consideration is that signal-to-noise ratio can influence the level of coherence in noisy signals. In the case of intermuscular coherence, the EMG can be considered noisy, since the coherent EMG component is a relatively low proportion of the total EMG signal power. However, while there were increases in fat-free mass (i.e., muscle mass) due to training, there were no differences between groups. Furthermore, since no changes in fat mass of the thigh region were observed in either group, morphological changes leading to differences in signal-to-noise ratio are unlikely to play a major role in our results. Indeed, when measured, there were no differences in signal-to-noise ratio between the age groups in the present study (data not shown).

### Effects of Strength Training

Maximum voluntary contraction increased as expected during the present study in both young and older individuals, although YOUNG gained more strength than OLDER due to the training. Most previous studies have shown equivalent strength gains between age-groups but some show greater gains in young people (Suetta et al., 2009; Greig et al., 2011). Therefore, our findings are not without precedent. Neither YOUNG nor OLDER improved steadiness during 20% MVC trials, and YOUNG reduced steadiness during 70% MVC trials. These results were unexpected since previous short-term strength training studies have observed improvements in steadiness in hand (Griffin et al., 2009) and knee extensor muscles (Tracy and Enoka, 2006). However, both YOUNG and OLDER showed remarkably low-force coefficient of variance (<1% at 20% MVC and <2% at 70% MVC) in comparison with the results of Tracy and Enoka (2006) already at pre-training. This would naturally reduce the likelihood of identifying a possible functional role of coherence on force steadiness.

If it is assumed that intermuscular coherence at about 10 Hz is reflective of Ia afferent feedback (Lippold, 1970; Erimaki and Christiakos, 2008), then there appears to be no training-induced changes in pre- or post-synaptic inhibition in either YOUNG or OLDER in the present study. Previous strength training studies have not seen consistent increases in H-reflex amplitude of the lower limbs, with reports of both positive (Aagaard et al., 2002) and no effect (Vila-Cha et al., 2012). Limited evidence suggests that afferent feedback from the lower limb is not improved by short-term strength training in older individuals (Unhjem et al., 2015), which could be of functional importance (e.g., in balance control). Nevertheless, it is difficult to ascertain possible mechanisms of adaptation.

An interesting finding is that 40–60 Hz coherence increased after training in YOUNG during 70% MVC trials but not in 100% MVC trials. The so-called Piper rhythm has been observed during high-force contractions (Piper, 1907) and has been observed in corticomuscular coherence studies (Brown et al., 1998), although not all lower limb muscles demonstrate a shift toward higher frequencies (Ushiyama et al., 2012). One feature that distinguished the 70% MVC trials from the 100% MVC trials in the present study was the intention to maintain force steadiness. During the 100% MVC trials, the subjects were instructed to exert their maximum force, and were not interested in keeping to a specific target. In this regard, there may be some other factor responsible for the training-induced increase in 40–60 Hz coherence observed during 70% MVC trials rather than simply a greater contraction force. It has been suggested that heightened attentiveness may possibly affect cortical Piper activity (DeFrance and Sheer, 1988; Brown et al., 1998). Since YOUNG showed worsening of force steadiness during 70% MVC trials, which were related to the magnitude of increased maximum strength, this group may have experienced adaptations that allow/require greater attentiveness to a high-force steadiness task.

## Conclusion

The present study identified clearly stronger intermuscular coherence in healthy young than older individuals during both low- and high-force isometric knee extension contractions, particularly over 8–40 Hz frequencies. A short-term strength training intervention increased the coherence strength during 70% MVC trials in young individuals (40–60 Hz) and reduced coherence strength during 100% MVC trials in older (8–14 Hz) individuals. It appears that enhanced 40–60 Hz coherence does not solely reflect the degree of force production capacity, since coherence level increased in YOUNG during 70% but not 100% MVC trials after training. Finally, the functional significance of intermuscular coherence level remains unclear, since neither the performance measures (i.e., force steadiness and maximum force) or their training-induced changes associated with the coherence/changes in coherence strength of any studied frequency band.

## Author Contributions

SW, JA, JW, RM, HP, and TP conceived and designed the study. SW performed the experiments. SW and SB analyzed the data. SW, RM, HP, and SB interpreted the results. SW and SB prepared the figures. SW drafted the manuscript. SW, JA, JW, RM, HP, SB, and TP edited and approved the final manuscript.

## Conflict of Interest Statement

The authors declare that the research was conducted in the absence of any commercial or financial relationships that could be construed as a potential conflict of interest.
